# Using the neutrophil‐to‐lymphocyte ratio to predict the outcome of individuals with nonsquamous non‐small cell lung cancer receiving pembrolizumab plus platinum and pemetrexed

**DOI:** 10.1111/1759-7714.15036

**Published:** 2023-07-19

**Authors:** Hisao Imai, Satoshi Wasamoto, Takeshi Tsuda, Yoshiaki Nagai, Takayuki Kishikawa, Ken Masubuchi, Takashi Osaki, Yosuke Miura, Yukihiro Umeda, Akihiro Ono, Hiroyuki Minemura, Yutaka Yamada, Junichi Nakagawa, Yuki Kozu, Hirokazu Taniguchi, Hiromitsu Ohta, Takashi Kasai, Kyoichi Kaira, Hiroshi Kagamu

**Affiliations:** ^1^ Department of Respiratory Medicine Comprehensive Cancer Center, International Medical Center, Saitama Medical University Hidaka Saitama Japan; ^2^ Division of Respiratory Medicine Gunma Prefectural Cancer Center Ota Gunma Japan; ^3^ Division of Respiratory Medicine Saku Central Hospital Advanced Care Center Saku Nagano Japan; ^4^ Division of Respiratory Medicine Toyama Prefectural Central Hospital Toyama Toayama Japan; ^5^ Department of Respiratory Medicine Jichi Medical University, Saitama Medical Center Saitama Saitama Japan; ^6^ Division of Thoracic Oncology Tochigi Cancer Center Utsunomiya Tochigi Japan; ^7^ Division of Respiratory Medicine National Hospital Organization Shibukawa Medical Center Shibukawa Gunma Japan; ^8^ Division of Allergy and Respiratory Medicine, Integrative Centre of Internal Medicine Gunma University Hospital Maebashi Gunma Japan; ^9^ Third Department of Internal Medicine Faculty of Medical Sciences, University of Fukui Eiheiji Fukui Japan; ^10^ Division of Internal Medicine Kiryu Kosei General Hospital Kiryu Gunma Japan; ^11^ Department of Pulmonary Medicine Fukushima Medical University Fukushima Fukushima Japan; ^12^ Division of Respiratory Medicine Ibaraki Prefectural Central Hospital Kasama Ibaraki Japan; ^13^ Division of Respiratory Medicine National Hospital Organization Takasaki General Medical Center Takasaki Gunma Japan

**Keywords:** body mass index, immune checkpoint inhibitors, neutrophil‐to‐lymphocyte ratio, nonsquamous non‐small cell lung cancer

## Abstract

**Background:**

Factors predicting the response to pembrolizumab plus platinum and pemetrexed combination therapy (Pemb‐Plt‐PEM) in nonsquamous non‐small cell lung cancer (non‐sq NSCLC) are unclear. We investigated the Glasgow Prognostic (GP) score, neutrophil‐to‐lymphocyte ratio (NLR), and body mass index (BMI) as predictors of response to initial treatment with combination therapy in individuals with advanced non‐sq NSCLC.

**Methods:**

We retrospectively reviewed 236 patients who received initial treatment with combination therapy for non‐sq NSCLC at 13 institutions between December 2018 and December 2020. The usefulness of the GP score, NLR, and BMI as prognostic indicators was assessed. Cox proportional hazard models and the Kaplan–Meier method were used to compare progression‐free survival (PFS) and overall survival (OS).

**Results:**

The response rate was 51.2% (95% CI: 44.9–57.5%). The median PFS and OS after beginning Pemb‐Plt‐PEM were 8.8 (95% CI: 7.0–11.9) months and 23.6 (95% CI: 18.7–28.6) months, respectively. The NLR independently predicted the efficacy of Pemb‐Plt‐PEM—the PFS and OS were more prolonged in individuals with NLR <5 than in those with NLR ≥5 (PFS: 12.8 vs. 5.3 months, *p* = 0.0002; OS: 29.4 vs. 12.0 months, *p* < 0.0001). BMI predicted the treatment response—individuals with BMI ≥22.0 kg/m^2^ had longer OS than did those with BMI < 22.0 kg/m^2^ (OS: 28.4 vs. 18.4 months, *p* = 0.0086).

**Conclusions:**

The NLR significantly predicted PFS and OS, whereas BMI predicted OS, in individuals who initially received Pemb‐Plt‐PEM for non‐sq NSCLC. These factors might be prognosis predictors in non‐sq NSCLC.

## Graphical abstract

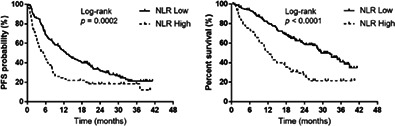

## INTRODUCTION

The highest number of deaths related to cancer is attributed to lung cancer; the death rate is higher for lung cancer than for colon, breast, or prostate cancer.^1^ Approximately 85%–90% of the cases are of non‐small cell lung cancer (NSCLC).^2^ Disease progression can be prevented and survival can be prolonged by using immune checkpoint inhibitors (ICIs) or both ICIs and cytotoxic chemotherapy drugs^3^ in treatment‐naive individuals with metastatic NSCLC.^4^, ^5^, ^6^, ^7^, ^8^

Pembrolizumab (Pemb), a monoclonal IgG4 antibody that targets the PD‐1 receptor, has been investigated for use in cancers, including NSCLC.^4^, ^5^ A global phase III trial (KEYNOTE‐189) assessed the use of Pemb plus platinum (Plt) and pemetrexed (PEM) combination therapy (Pemb‐Plt‐PEM), in terms of effectiveness and feasibility.^4^ Pemb‐Plt‐PEM prolonged progression‐free survival (PFS) and overall survival (OS) further than Plt plus PEM combination therapy did.^4^ A real‐world study of effectiveness of initial Pemb‐Plt‐PEM for nonsquamous NSCLC (non‐sq NSCLC) showed comparable results to those of the KEYNOTE‐189 study.^9^

Distant metastases are often present when NSCLC is diagnosed. In advanced stages, weight loss and systemic inflammatory responses (SIRs), including cancer cachexia, may occur.^10^, ^11^ Furthermore, the cancer‐related survival outcome is predicted by SIR‐based scoring systems, including the neutrophil‐to‐lymphocyte (NL) ratio and Glasgow Prognostic (GP) score. The GP score comprises the serum C‐reactive protein (CRP) and albumin levels;^10^ it predicts the prognosis in advanced NSCLC.^12^, ^13^, ^14^, ^15^ However, to date, no analyses have evaluated the potential of a relationship of the GP score with the response to initial ICI treatment combined with chemoimmunotherapy for non‐sq NSCLC.

Previous studies have reported that SIR‐based markers predict the treatment efficacy of ICIs. Indeed, the NL ratio predicted the outcome of ICI treatment in individuals with skin,^16^, ^17^, ^18^ kidney,^19^ or lung cancer.^20^, ^21^, ^22^, ^23^

Body mass index (BMI) might be a predictor of prognosis in malignancies. BMI is also used as a measure of sarcopenia, which correlates with adverse prognosis in individuals with NSCLC who receive ICIs.^24^ In addition, BMI correlates with the response to ICIs in solid tumors, including skin, kidney, and lung cancers.^25^ BMI has been associated with ICI treatment effectiveness in NSCLC.^26^ However, such an association is unknown in individuals with non‐sq NSCLC who initially received combined chemoimmunotherapy.

Currently, limited data are available regarding the relationship of the GP score, NL ratio, and BMI with the effectiveness of frontline combined chemoimmunotherapy for individuals with non‐sq NSCLC. Furthermore, first‐line Pemb‐Plt‐PEM is frequently used in patients with non‐sq NSCLC, but none of them have been studied. We aimed to evaluate whether these factors predicted the effectiveness of initial Pemb‐Plt‐PEM in individuals with non‐sq NSCLC.

## METHODS

### Participants

We retrospectively analyzed the efficacy of first‐line Pemb‐Plt‐PEM treatment for non‐sq NSCLC at 13 Japanese institutions (December 2018 to December 2020). The study participants (1) had non‐sq NSCLC at inoperable disease stage III/IV or postoperative recurrent disease, which was diagnosed with histological or cytological analysis and (2) had undergone initial Pemb‐Plt‐PEM treatment.

There were 248 consecutive individuals who were administered Pemb‐Plt‐PEM; among them, 11 with druggable driver gene mutations/translocations received molecular targeted therapy as first‐line treatment. One individual had a significant amount of missing data. Overall, 236 individuals were included in the study (Figure S1).

The 2015 World Health Organization system was used to classify NSCLC. The individuals underwent systematic evaluation and staging prior to treatment. The clinical stage was determined based on the tumor‐node‐metastasis (TNM) system^27^ and assigned based on physical examinations, chest radiography, computed tomography (CT) scans of the chest/abdomen, CT or magnetic resonance imaging of the brain, and bone scintigraphy/^18^F‐fluorodeoxyglucose positron‐emission tomography. Formalin‐fixed tumor specimens were used to determine PD‐L1 expression using a PD‐L1 immunohistochemistry kit (22C3 pharmDx assay; Dako).^28^ Demographic characteristics, clinical factors, and responses to Pemb‐Plt‐PEM were extracted from records. For each individual, a censored event or death was investigated for survival analysis.

The design of the current study was approved by the Institutional Ethics Committee of the International Medical Center, Saitama Medical University (approval no.: 2022‐036). The study followed the institutional and national ethical standards and the Declaration of Helsinki (2013 revision). No animal experiments were performed. Because of its retrospective design, patient informed consent was not obtained. However, the opt‐out method was available to refuse participation in the study.

### Treatment

Individuals with a history of receiving ICIs, including the Pemb‐Plt‐PEM regimen, were not present in the current analysis population. The basic therapeutic regimen consisted of Pemb (standard dose of intravenous 200 mg on day 1 of each cycle), intravenous cisplatin (75 mg/m^2^ body surface area) or carboplatin (area under the concentration–time curve, 5 mg/mL/min), according to the investigator's discretion, plus PEM (500 mg/m^2^) up to six cycles, all administered intravenously every 3 weeks, followed by PEM (500 mg/m^2^) and 200 mg of Pemb every 3 weeks. The premedication consisting of folic acid, vitamin B12, and glucocorticoids was administered based on each institution's treatment protocol. In some individuals, Pemb or PEM was omitted from the Pemb and PEM maintenance therapy, dependent on the treating physician's decision. Treatment was discontinued if progressive disease developed, irreversible toxicity was noted, or the individual withdrew their consent to receive anticancer therapy.

### Treatment efficacy evaluation

Serum concentrations of CRP and albumin were assessed on the day of, or the day before, Pemb‐Plt‐PEM treatment. GP scores were categorized as: 0–CRP <1.0 mg/dL and albumin ≥3.5 mg/dL; 1–only an increase in CRP or only a decrease in albumin concentration; and 2–CRP ≥1.0 mg/dL and albumin <3.5 mg/dL.

The NL ratio (i.e., absolute neutrophil count: absolute lymphocyte count) has thresholds of ≥5 and <5.^20^, ^22^, ^29^, ^30^, ^31^, ^32^, ^33^, ^34^, ^35^, ^36^, ^37^, ^38^, ^39^, ^40^ In the current analysis, we set a cutoff NL ratio of 5.0. Based on the NL ratio, low‐ (<5.0) and high‐ (≥5.0) risk individuals were identified.

BMI (weight [kg]/height [m^2^]) was assessed before the start of Pemb‐Plt‐PEM administration. We evaluated the potential correlation between BMI and Pemb‐Plt‐PEM effectiveness based on a BMI cutoff value of 22.0 kg/m^2^, that is, the ideal BMI for the Japanese population^41^ (high and low BMI: ≥22.0 and < 22.0 kg/m^2^, respectively).

The tumor treatment response was quantified based on the best overall response and maximum amount of tumor shrinkage. Furthermore, radiological tumor responses were evaluated based on the Response Evaluation Criteria in Solid Tumors (version 1.1).^42^ The PFS interval was determined from day 1 of Pemb‐Plt‐PEM administration until the first occurrence of disease progression or death from any cause. The OS interval was determined from day 1 of Pemb‐Plt‐PEM administration until death or censoring at the last follow‐up.

### Statistical analysis

Categorical and continuous variables were analyzed using Fisher's exact test and Welch's *t*‐test, respectively, in subgroups defined by the GP score, NL ratio, and BMI. Cox proportional hazard models with stepwise regression were used to evaluate factors predicting PFS and OS. The hazard ratios (HRs) with 95% confidence intervals (CIs) were calculated. Univariate and multivariable logistic regression analyses were undertaken for different outcomes. Kaplan–Meier survival analysis was performed to estimate survival, whereas survival was evaluated using the log‐rank test. Two‐tailed *p* < 0.05 indicated statistical significance. Statistical analyses were performed using JMP software for Windows, version 11.0 (SAS Institute).

## RESULTS

### Baseline factors and tumor responses

Table 1 describes the demographic factors of the study participants (*n* = 236). Table 2 presents the treatment responses. The response and disease control rates were 51.2% (95% CI: 44.9–57.5) and 81.3% (95% CI: 75.8–85.8), respectively.

**TABLE 1:** Patient characteristics.

Characteristics	Total number of patients (*n* = 236)
Sex	
Male/female	189/47
Median age at treatment (years) (range)	68 (24–82)
Performance status	
0/1/2/3/4	82/138/15/1/0
Smoking history	
Yes/no	209/27
Clinical stage at diagnosis	
II/III/IV/postoperative recurrence	1/9/178/48
Histology	
Adenocarcinoma/others	216/20
PD‐L1 tumor proportion score (%)	
<1/1–49/≥50/unknown	77/75/57/27
Driver gene mutation/translocation	
*EGFR*/*ALK*/others/wild‐type, negative, or unknown^a^	5/0/27/204
History of postoperative adjuvant chemotherapy	
Yes/no	33/203
Intracranial metastases at initial treatment	
Yes/no	58/178
Liver metastases at initial treatment	
Yes/no	12/224
Bone metastases at initial treatment	
Yes/no	88/148
Body mass index (kg/m^2^)	
Median (range)	22.2 (13.3–36.5)
Prior radiotherapy^b^	
Yes/no	58/178
Number of cycles of platinum+pemetrexed+pembrolizumab administered	
Median	4
Range	1–6
Number of cycles of maintenance therapy administered^c^	
Median	3
Range	0–51
Platinum agent	
Cisplatin/carboplatin	46/190
Reason for discontinuation of platinum + pemetrexed + pembrolizumab administration^d^	
Progressive disease	28
Adverse events	39
Worsening of performance status	6
Others	9
Steroid treatment for adverse events^e^	
Yes/no	63/173
Laboratory data, median (range)	
C‐reactive protein (mg/dL)	0.70 (0.01–21.0)
Albumin (g/dL)	3.7 (1.7–4.8)
Neutrophil (cells/μL)	4718.5 (1200–23 360)
Lymphocyte (cells/μL)	1361 (285–3610)
Continuing administration of maintenance therapy at data cutoff	23

^a^ Test results showed no known genetic abnormalities such as *EGFR* mutations and *ALK* fusion genes, or no known genetic abnormalities had been tested for.

^b^ Curative intent and palliative radiotherapy.

^c^ Including pemetrexed + pembrolizumab, pemetrexed, or pembrolizumab maintenance therapy.

^d^ Excluding maintenance therapy.

^e^ Excluding topical agents.

**TABLE 2:** Treatment response.

	Total (*n* = 236)
Treatment response	
CR	11
PR	110
SD	71
PD	38
NE	6
Response rate (%) (95% CI)	51.2 (44.9–57.5)
Disease control rate (%) (95% CI)	81.3 (75.8–85.8)

Abbreviations: CR, complete response; NE, not evaluated; PD, progressive disease; PR, partial response; SD, stable disease.

### Comparisons of predictors between groups

The characteristics of the GP score, NL ratio, and BMI subgroups are demonstrated in Table 3. The pretreatment GP score was 0–1 (164 individuals) or 2 (72 individuals). The performance status (PS), bone metastases at initial treatment, BMI, serum CRP and albumin levels, neutrophil count, and disease control rate were related to the GP score (all *p* < 0.05).

**TABLE 3:** Patient characteristics based on GPS, NLR, and BMI.

Variables	GPS			NLR			BMI		
	0–1	2	*p*‐value	Low (<5)	High (≥5)	*p*‐value	Low (<22.0)	High (≥22.0)	*p*‐value
Patients (*n*)	164	72		160	76		114	122	
Characteristics									
Sex									
Male/female	130/34	59/13	0.72	126/34	63/13	0.49	84/30	105/17	**0.02**
Median age at treatment (years) (range)	68.5 (24–82)	66.5 (34–81)	0.19^a^	69 (37–82)	65.5 (24–81)	**0.007** ^a^	68 (24–81)	68 (37–82)	0.75^a^
Performance status (PS)									
0–1/≥ 2	158/6	62/10	**0.008**	153/7	67/9	**0.04**	105/9	115/7	0.6
Smoking history									
Yes/no	141/23	68/4	0.07	141/19	68/8	0.83	100/14	109/13	0.83
Intracranial metastases at initial treatment									
Yes/no	37/127	21/51	0.32	35/125	23/53	0.19	27/87	31/91	0.76
Liver metastases at initial treatment									
Yes/no	9/155	3/69	>0.99	7/153	5/71	0.53	3/111	9/113	0.13
Bone metastases at initial treatment									
Yes/no	51/113	37/35	**0.003**	51/109	37/39	**0.014**	44/70	44/78	0.78
BMI (kg/m^2^)									
Median (range)	22.8 (13.3–36.5)	21.0 (15.6–28.6)	**0.0002** ^a^	22.7 (13.3–36.5)	21.0 (15.1–28.8)	**0.0024** ^a^	20.1 (13.3–21.9)	24.5 (22.0–36.5)	‐
Prior radiotherapy^‡^									
Yes/no	38/126	20/52	0.51	28/132	30/46	**0.0004**	33/81	25/97	0.17
Administration cycles of pembrolizumab plus platinum and pemetrexed									
Median (range)	4 (1–5)	4 (1–6)	0.25^a^	4 (1–4)	4 (1–6)	**0.021** ^a^	4 (1–6)	4 (1–5)	0.37^a^
Administration cycles of maintenance therapy									
Median (range)	4 (0–47)	3 (0–51)	0.09^a^	4 (0–47)	2 (0–51)	0.15†	3 (0–51)	4 (0–48)	**0.03** ^a^
Laboratory data									
CRP (mg/dL)	0.28	5.48	**<0.0001** ^a^	0.35	3.4	**<0.0001** ^a^	1.22	0.35	**0.013** ^a^
Albumin (g/dL)	3.9	3	**<0.0001** ^a^	3.8	3.3	**<0.0001** ^a^	3.4	3.9	**<0.0001** ^a^
Neutrophil (cells/μL)	4288	6206	**<0.0001** ^a^	4159	7222	**<0.0001** ^a^	5200	4565	0.09^a^
Lymphocyte (cells/μL)	1411	1201	0.06^a^	1600	935	**<0.0001** ^a^	1195	1515	**0.0039** ^a^
Treatment response									
CR	8	3		9	2		7	4	
PR	75	35		77	33		48	62	
SD	57	14		52	19		33	38	
PD	21	17		20	18		23	15	
NE	3	3		2	4		3	3	
Response rate (%) (95% CI)	50.6 (43.0–58.1)	52.7 (41.3–63.8)	0.770	53.7 (46.0–61.2)	46.0 (35.3–57.1)	0.32	48.2 (39.2–57.3)	54.0 (45.2–62.6)	0.43
Disease control rate (%) (95% CI)	85.3 (79.0–90.0)	72.2 (60.8–81.2)	**0.028**	86.2 (79.9–90.8)	71.0 (59.9–80.0)	**0.0071**	77.1 (68.6–83.9)	85.2 (77.7–90.5)	0.13

*Note*: Fisher's exact test. Bold font indicates a statistically significant difference.

Abbreviations: BMI, body mass index; CI, confidence interval; CR, complete response; CRP, C‐reactive protein; GPS, Glasgow prognostic score; NE, not evaluated; NLR, neutrophil‐to‐lymphocyte ratio; PD, progressive disease; PR, partial response; SD, stable disease.

^a^ Welch's *t*‐test.

^b^ Including palliative radiotherapy and curative intent chemoradiotherapy.

The NL ratio at the start of Pemb‐Plt‐PEM administration was categorized as low (160 individuals) or high (76 individuals). The median age at treatment, PS, bone metastases at the start of treatment, BMI, prior radiotherapy, number of Pemb‐Plt‐PEM administration cycles, serum CRP and albumin levels, neutrophil and lymphocyte counts, and disease control rate were related to the NL ratio values (all *p* < 0.05).

The pretreatment BMI was low and high in 114 and 122 individuals, respectively. Sex and the number of maintenance therapy administration cycles, serum CRP level, albumin concentration, and lymphocyte count were related to the BMI (all *p* < 0.05).

### Treatment efficacy for survival

**FIGURE 1:**
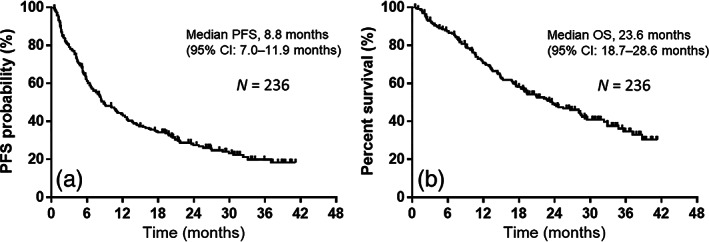
Kaplan–Meier curves for progression‐free survival (PFS) and overall survival (OS). (a) The median PFS was 8.8 months among all 236 individuals who received pembrolizumab plus platinum and pemetrexed as first‐line treatment. (b) The median OS was 23.6 months among the 236 individuals who received pembrolizumab plus platinum and pemetrexed as first‐line treatment.

The median PFS and OFS were 8.8 (95% CI: 7.0–11.9) months (Figure 1a) and 23.6 (95% CI: 18.7–28.6) months (Figure 1b), respectively, after a median follow‐up duration of 18.7 (range, 0.5–41.3) months. At the data cutoff date (June 30, 2022), 129 of 236 individuals had died, and 107 had survived. Table 4 summarizes the results of the univariate and multivariable analyses for PFS and OS. Univariate analyses indicated associations of PFS with the PD‐L1 tumor proportion score (TPS), bone metastases at initial treatment, use of prior radiotherapy, and NL ratio. Additionally, univariate analyses indicted associations of OS with the PS, PD‐L1 TPS, bone metastases at initial treatment, GP score, NL ratio, and BMI.

**TABLE 4:** Univariate and multivariable analyses of factors for PFS and OS.

Variables	Median PFS	Univariate analysis			Multivariable analysis			Median OS	Univariate analysis			Multivariable analysis		
	(months)	HR	95% CI	*p*‐value	HR	95% CI	*p*‐value	(months)	HR	95% CI	*p*‐value	HR	95% CI	*p*‐value
Sex														
Male/female	9.1/8.0	1.06	0.72–1.59	0.76				24.6/18.9	0.96	0.63–1.54	0.88			
Age														
<75/≥75	9.0/7.5	0.82	0.54–1.32	0.41				23.6/24.0	0.85	0.52–1.48	0.56			
Performance status (PS)														
0–1/2–3	9.0/3.1	0.57	0.90–2.97	0.09	0.51	0.28–1.00	0.05	24.3/9.7	0.43	0.24–0.83	**0.014**	0.46	0.25–0.90	**0.027**
Smoking history														
Yes/No	9.0/6.6	0.85	0.55–1.38	0.50				24.3/17.5	0.77	0.47–1.36	0.36			
PD‐L1 tumor proportion score (%)														
<50 or unknown/≥ 50	7.7/19.2	2	1.37–3.01	**0.0002**	2.81	1.86–4.39	**<0.0001**	19.4/38.7	1.72	1.12–2.75	**0.0112**	2.89	1.81–4.82	**<0.0001**
Intracranial metastases at initial treatment														
Yes/No	6.8/8.8	0.85	0.58–1.20	0.36				18.8/23.7	1.04	0.69–1.54	0.82			
Liver metastases at initial treatment														
Yes/No	5.8/8.8	1.4	0.69–2.52	0.32				13.7/24.0	1.59	0.71–3.05	0.23			
Bone metastases at initial treatment														
Yes/No	6.4/11.2	1.42	1.04–1.93	**0.026**	1.39	1.00–1.91	**0.0472**	14.8/28.6	1.78	1.25–2.52	**0.0014**	1.59	1.09–2.31	**0.0161**
Prior radiotherapy														
Yes/No	10.9/8.8	0.93	0.64–1.31	0.71				19.3/23.6	0.96	0.63–1.43	0.87			
GPS														
0, 1/2	11.2/6.6	0.72	0.53–1.01	0.05	0.82	0.57–1.21	0.32	28.6/12.4	0.54	0.37–0.78	**0.0012**	0.72	0.47–1.10	0.13
NLR														
Low (<5)/High (≥ 5)	12.8/5.3	0.55	0.40–0.76	**0.0004**	0.57	0.40–0.81	**0.002**	29.4/12.0	0.41	0.28–0.58	**<0.0001**	0.47	0.31–0.70	**0.0003**
BMI (kg/m^2^)														
Low (<22.0)/High (≥ 22.0)	6.8/11.2	1.33	0.98–1.80	0.06	1.25	0.91–1.72	0.16	18.4/28.4	1.58	1.12–2.25	**0.009**	1.46	1.01–2.10	**0.0391**

*Note*: Bold font indicates a statistically significant difference.

Abbreviations: BMI, body mass index; CI, confidence interval; GPS, Glasgow prognostic score; HR, hazard ratio; NLR, neutrophil‐to‐lymphocyte ratio; OS, overall survival; PD‐L1, programmed cell death ligand 1; PFS, progression‐free survival.

The multivariable analysis demonstrated that PD‐L1 TPS < 50% (HR: 2.81, *p* < 0.0001) and bone metastases at initial treatment (HR: 1.39, *p* = 0.0472) were related to worse PFS, whereas a low (<5) NL ratio was related to prolonged PFS (HR: 0.57, *p* = 0.002). The multivariable analyses also showed that a PS of 0–1 (HR: 0.46, *p* = 0.027) and low (<5) NL ratio (HR: 0.47, *p* = 0.0003) correlated with better OS, whereas PD‐L1 TPS < 50% (HR: 2.89, *p* < 0.0001), bone metastases at initial treatment (HR: 1.59, *p* = 0.0161), and low (<22.0 kg/m^2^) BMI (HR: 1.46, *p* = 0.0391) correlated with shorter OS.

**FIGURE 2:**
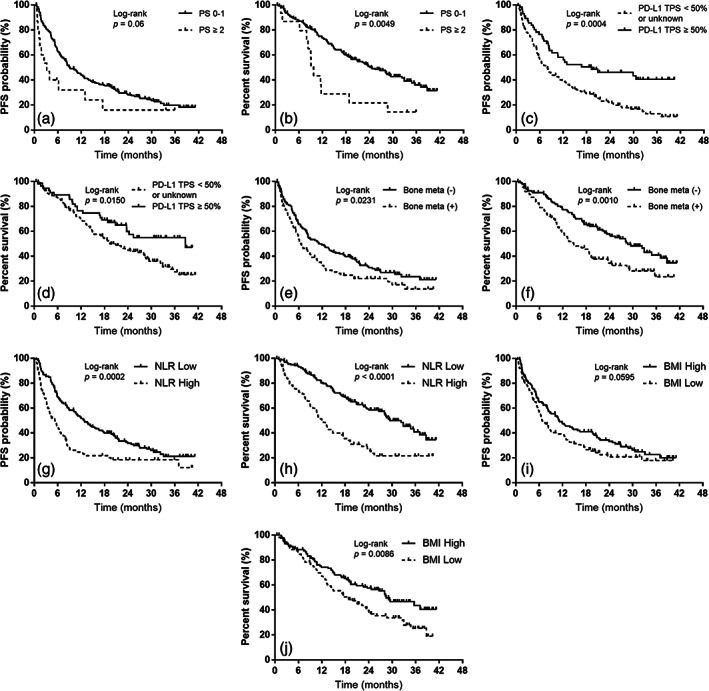
Kaplan–Meier curves for progression‐free survival (PFS) and overall survival (OS) according to performance status (PS) at the start of pembrolizumab plus platinum and pemetrexed treatment, programmed death ligand‐1 tumor proportion score (PD‐L1 TPS), presence of bone metastases at initial treatment, neutrophil‐to‐lymphocyte (NL) ratio, and body mass index (BMI). (a) PFS according to the PS at the start of pembrolizumab plus platinum and pemetrexed treatment (PS 0–1, median PFS: 9.0 months; PS ≥2, median PFS: 3.1 months). (b) OS according to the PS at the start of pembrolizumab plus platinum and pemetrexed treatment (PS 0–1, median OS: 24.3 months; PS ≥2, median OS: 9.7 months). (c) PFS according to PD‐L1 TPS (PD‐L1 TPS ≥50%, median PFS: 19.2 months; PD‐L1 TPS < 50% or unknown, median PFS: 7.7 months). (d) OS according to PD‐L1 TPS (PD‐L1 TPS ≥50%, median OS: 38.7 months; PD‐L1 TPS < 50% or unknown, median OS: 19.4 months). (e) PFS according to the presence of bone metastases at initial treatment (without bone metastases at initial treatment, median PFS: 11.2 months; with bone metastases at initial treatment, median PFS: 6.4 months). (f) OS according to the presence of bone metastases at initial treatment (without bone metastases at initial treatment, median OS: 28.6 months; with bone metastases at initial treatment, median OS: 14.8 months). (g) PFS according to the NL ratio (NL ratio <5, median PFS: 12.8 months; NL ratio ≥5, median PFS: 5.3 months). (h) OS according to the NL ratio (NL ratio <5, median OS: 29.4 months; NL ratio ≥5, median OS: 12.0 months). (i) PFS according to BMI (BMI ≥22.0, median PFS: 11.2 months; BMI < 22.0, median PFS: 6.8 months). (j) OS according to BMI (BMI ≥22.0, median OS: 28.4 months; BMI < 22.0, median OS: 18.4 months).

PFS and OS survival curves were constructed using Kaplan–Meier analysis (Figure 2). Although a PS of 0–1 indicated a trend toward better PFS (9.0 months), which was not statistically significant, the PFS was similar to that of individuals with a PS ≥2 (3.1 months) (*p* = 0.06; Figure 2a). Nevertheless, OS was more prolonged with a PS of 0–1 (24.3 months) than with that of ≥2 (9.7 months; *p* = 0.0049; Figure 2b). Individuals with PD‐L1 TPS ≥50% had a prolonged median PFS (19.2 months) compared with that of individuals with PD‐L1 TPS < 50% or unknown (7.7 months; log‐rank test, *p* = 0.0004; Figure 2c). Similarly, OS was prolonged in individuals with PD‐L1 TPS ≥50% (38.7 months) compared with that in individuals with PD‐L1 TPS < 50% or unknown (19.4 months; log‐rank test, *p* = 0.0150; Figure 2d). Individuals without pretreatment bone metastases had prolonged median PFS (11.2 months) compared with individuals with bone metastases (6.4 months; log‐rank test, *p* = 0.0231; Figure 2e). Moreover, OS was longer in individuals lacking bone metastases (28.6 months) than in individuals with bone metastases (14.8 months; log‐rank test, *p* = 0.0010; Figure 2f). An NL ratio <5 was associated with a more prolonged median PFS (12.8 months) than was an NL ratio ≥5 (5.3 months; log‐rank test, *p* = 0.0002; Figure 2g). Similarly, the NL ratio <5 group had a longer OS (29.4 months) than did the NL ratio ≥5 group (12.0 months; log‐rank test, *p <* 0.0001; Figure 2h). Although individuals with BMI ≥22.0 kg/m^2^ tended to have better PFS (11.2 months), it was not significantly different between them and those with BMI < 22.0 kg/m^2^ (6.8 months; *p* = 0.0595; Figure 2i). However, OS was significantly more prolonged with BMI ≥22.0 kg/m^2^ (28.4 months) than with BMI < 22.0 kg/m^2^ (18.4 months; *p* = 0.0086; Figure 2j).

## DISCUSSION

The current analysis assessed relationships among the GP score, NL ratio, and BMI and therapeutic effectiveness of initial Pemb‐Plt‐PEM treatment for individuals with advanced non‐sq NSCLC. Consequently, multivariable analyses demonstrated that the NL ratio and BMI were independently related to OS, indicating they might predict OS after initial Pemb‐Plt‐PEM treatment for advanced non‐sq NSCLC. To the best of our knowledge, no previous study has assessed associations among the GP score, NL ratio, and BMI and the survival of individuals with advanced non‐sq NSCLC who initially received combined chemoimmunotherapy.

The GP score is determined using CRP and albumin levels; these are conveniently determined in the clinic.^10^ Several studies have shown associations between the GP score and efficacies of various ICIs, in different treatment lines, for individuals with NSCLC and various PD‐L1 expression levels.^15^, ^43^, ^44^ The univariate analysis for PFS demonstrated an insignificant trend toward longer PFS for individuals with GP scores 0–1 than for those with GP scores 2. The OS did not differ between individuals with a GP score of 0–1 or 2. The reasons for these findings are uncertain and should be studied in the future. It remains unknown whether these results are limited to the first‐line use of Pemb‐Plt‐PEM or if the combination of a cytotoxic drug plus Pemb should be considered separately from Pemb in individuals with NSCLC and high PD‐L1 expression levels.

The NL ratio has demonstrated prognostic applicability across multiple tumor types.^45^ Previous studies of NSCLC have determined the prognostic ability of the baseline NL ratio.^46^, ^47^ In addition, systematic reviews demonstrated that the NL ratio predicts treatment effectiveness and outcomes in NSCLC.^48^ Some reports have indicated that the NL ratio predicts the prognosis of individuals, but the results are contradictory across reports. In our analysis, patient characteristics and the NL ratio had an association with previous radiotherapy, suggesting a confounding effect of clinical factors. Hematological parameters are commonly and easily obtained in clinical practice.^49^ Furthermore, the NL ratio reflects host immune reactions and inflammation and is related to a poor prognosis for individuals with NSCLC who receive immunotherapy.^50^ The NL ratio indicates systemic inflammation and the immune system balance under malignant biological conditions.^51^, ^52^ Notably, neutrophils secrete immunosuppressive and angiogenic factors that promote a protumor microenvironment.^53^ Additionally, low numbers of circulating lymphocytes likely result in fewer tumor‐infiltrating lymphocytes (TILs) and low antitumor T cell responses.^54^ Petrova et al. demonstrated that neutrophils and platelets promote tumor development and progression via the secretion of cytokines and chemokines, including MMP, IL‐6, IL‐8, TGF‐β, and VEGF,^55^ all of which can affect tumor cells indirectly or directly via the tumor microenvironment. Additionally, neutrophils participate in inflammatory responses that inhibit antitumor immune responses by suppressing cytotoxic CD8^+^ T cells. Moreover, in recent studies, a high neutrophil count and low lymphocyte count correlated with poor survival outcomes.^50^, ^55^ Although several studies have shown that changes in the NL ratio before and after treatment initiation could be used to assess treatment efficacy,^56^ we evaluated the NL ratio at the start of treatment but did not examine dynamic changes in the NL ratio after treatment initiation. Various cutoff values for the NL ratio along with the types of immunotherapies, PFS, and OS are summarized in Table S1. An NL ratio of five was the commonest adopted cutoff value previously^22^, ^29^, ^30^, ^37^, ^38^, ^57^ and the most appropriate value for Western countries; therefore, it was recommended for clinical application.^58^ The current analysis suggests that PFS and OS were shorter with an NL ratio ≥5 than with an NL ratio <5, which agrees with the findings described by Mei et al.^59^ Although the threshold was not definitively established, it appears generally acceptable to adopt five as an NL ratio cutoff value for prognostic determination.

A large retrospective cohort study suggested that a high BMI was related to better PFS and OS after ICI treatment of advanced melanoma.^60^ Another analysis showed that BMI was related to ICI effectiveness in solid malignancies, including melanoma, renal cell carcinoma, and NSCLC.^25^ Additionally, another study demonstrated a relationship between the BMI and ICI clinical efficacies in NSCLC,^26^ and BMI significantly correlated with ICI effectiveness in individuals with NSCLC who received second‐ or later‐line PD‐1/PD‐L1 blockade therapy. Tateishi et al. treated individuals with ICI monotherapy as first‐line and second‐ or later‐line treatments and demonstrated similar PD‐1 inhibitor efficacy between overweight and nonoverweight individuals (BMI ≥25 and < 25 kg/m^2^, respectively).^61^ However, those studies only included ICI monotherapy and did not include combination therapy with cytotoxic anticancer drugs. Moreover, in a previous report, carboplatin‐based combination chemotherapy that did not contain ICIs improved PFS and OS of overweight individuals compared with those of underweight individuals.^62^ In our study, patient characteristics did not differ between high‐ and low‐BMI individuals, except for sex, the number of maintenance therapy cycles administered, CRP and albumin levels, and lymphocyte counts. The response and disease control rates were similar between low‐ and high‐BMI individuals; however, BMI predicted OS but not PFS. Thus, a higher BMI might improve the survival benefit conferred by Pemb‐Plt‐PEM in these individuals and might allow individuals to receive subsequent treatments after progressive disease. We previously demonstrated that the BMI independently predicted the survival outcome of individuals with NSCLC expressing high PD‐L1 (PD‐L1 TPS ≥50%) who were treated with initial Pemb monotherapy; overweight individuals had prolonged survival, but not PFS, compared with underweight individuals.^44^ Our study included individuals with known and unknown levels of PD‐L1 expression, although all individuals received first‐line treatment. In this analysis, the reason for the longer OS in individuals with higher BMI may be the association of BMI with longer survival in individuals with NSCLC and high PD‐L1 expression levels who initially receive Pemb monotherapy, as previously described. However, in the present analysis, pembrolizumab was combined with a cytotoxic anticancer drug, and the PD‐L1 expression status was not high expression only; therefore, we were unable to reach a definitive conclusion. We set the BMI threshold at 22 kg/m^2^, that is, the ideal BMI for Japanese individuals; however, whether this is an appropriate cutoff value should be investigated in a future analysis, given the presence of variance related to differences in ethnicities and populations. Furthermore, BMI is influenced by various factors, including natural body size, genetic factors, tumor progression, the presence of cachexia, and psychological factors. We suspect that BMI contains many confounding factors. Therefore, even with multivariable analysis, it is difficult to identify the relationship between BMI and treatment efficacy or OS independently.

Our study has some weaknesses. First, this retrospective study depended on subjective assessments of tumor responses that might have led to errors in the recorded data related to treatment responses and PFS. Second, as there are no absolutely established cutoff values for the GP score, NL ratio, and BMI, we used cutoff values from previous studies. It will be important to examine whether the findings of the current analysis are clinically appropriate for other and larger cohorts in the future.

In summary, our findings suggest that the NL ratio is independently correlated with PFS and OS. Additionally, BMI is independently correlated with OS. Large‐scale studies should examine the generalizability of our findings. Although future analyses are required to confirm these results, our findings indicate that determining the NL ratio and BMI may help predict the efficacy and prognosis of individuals with advanced non‐sq NSCLC treated with initial Pemb‐Plt‐PEM treatment.

## AUTHOR CONTRIBUTIONS

All authors have approved the final manuscript. All authors had full access to the data in the study and take responsibility for the integrity of the data and the accuracy of the data analysis. *Conceptualization and methodology*, H.I. and K.K. (Kaira); *Formal analysis and data curation*, H.I. and K.K. (Kaira); *Project administration, visualization, and writing—original draft preparation*, H.I.; *Supervision*, K.K. (Kaira) and H.K.; *Investigation and resources*, S.W., T.T., Y.N., T.K., K.M., T.O., Y.M., Y.U., A.O., H.M., Y.Y., J.N., Y.K., H.T., H.O., and T.K.; *Writing—review and editing*, all authors.

## CONFLICT OF INTEREST STATEMENT

No potential conflict of interest exits.

## Supporting information


**Data S1.** Supporting Information
