# Supplementary material for: Using the neutrophil‐to‐lymphocyte ratio to predict the outcome of individuals with nonsquamous non‐small cell lung cancer receiving pembrolizumab plus platinum and pemetrexed
Source: Thorac Cancer. 2023 Jul 19;14(25):2567–78. doi: 10.1111/1759-7714.15036 (PMC10481141; doi:10.1111/1759-7714.15036)
Supplement: Supplementary file 1 — Data S1. Supporting Information [file TCA-14-2567-s001.docx]

Supplementary Figure 1. Diagram showing patient selection. The patients were treated with pembrolizumab plus platinum and pemetrexed between December 2018 and December 2020.


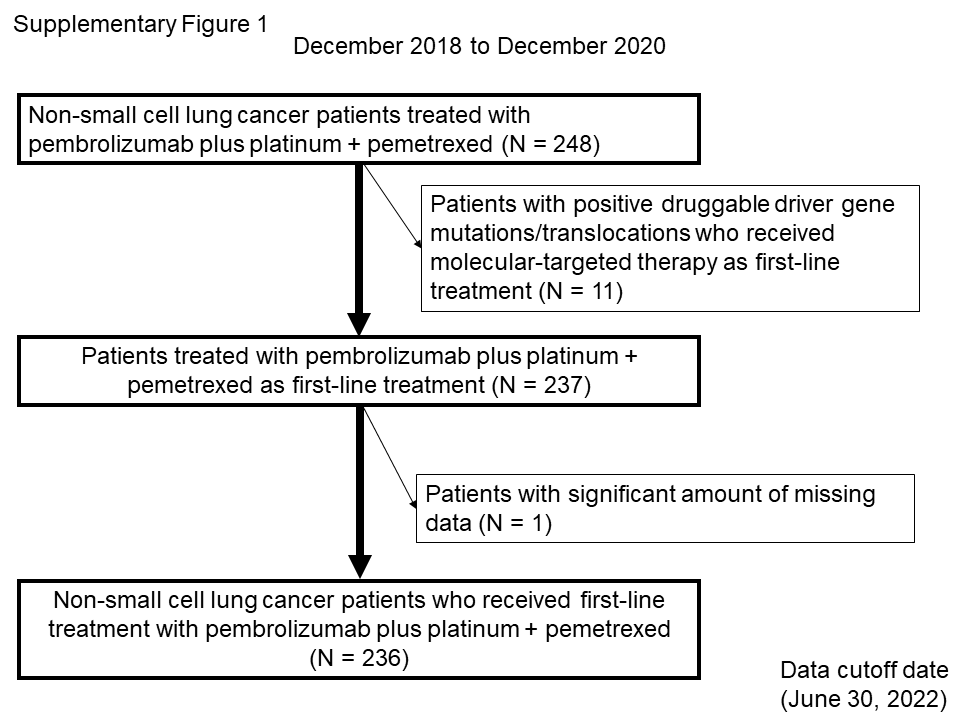


Supplementary Table 1. Baseline characteristics, NLR cut-off values, types of immunotherapies, PFS, and OS in previous reports on NLR

| Reference No. | Author | Sample size | Histology of NSCLC | Types of immunotherapies | NLR cut-off value | PFS, NLR-low vs NLR-high; *p*-value | OS, NLR-low vs NLR-high; *p*-value |
| --- | --- | --- | --- | --- | --- | --- | --- |
| [22] | Suh et al. | 54 | Adenocarcinoma, squamous cell carcinoma, adenosquamous cell carcinoma, NOS, pleomorphic carcinoma, large cell carcinoma | Nivolumab or pembrolizumab | 5 | 6.1 months/  1.3 months; *p* < 0.001 | 14.0 months/  2.1 months;  *p* < 0.001 |
| [29] | Takeda et al. | 30 | Adenocarcinoma, squamous cell carcinoma | Nivolumab | 5 | 95 days/  10 days; N/A | N/A |
| [30] | Khunger et al. | 109 | Adenocarcinoma, squamous cell carcinoma, others | Nivolumab | 5 | N/A | 29.1 months/  24.2 months;  *p* < 0.001 |
| [31] | Zer et al. | 88 | Adenocarcinoma, squamous cell carcinoma, large cell carcinoma, others | PD-1/PD-L1 blockade | 4 | N/A | 21.4 months/  6.8 months;  *p* = 0.019 |
| [32] | Svaton et al. | 120 | Adenocarcinoma, squamous cell carcinoma | Nivolumab | 3.8 | 6.1 months/  4.1 months; *p* = 0.321 | 14.2 months/  9.2 months;  *p* = 0.02 |
| [33] | Nakaya et al. | 101 | Squamous cell carcinoma, non-squamous cell carcinoma | Nivolumab | 3 | 5.3 months/  2.01 months;  *p* = 0.00515 | N/A |
| [34] | Pavan et al. | 184 | Adenocarcinoma, squamous cell carcinoma, NOS, sarcomatoid | Nivolumab, pembrolizumab, or atezolizumab | 3 | 7.4 months/  3.1 months; N/A | 49.3 months/  17.3 months; N/A |
| [35] | Prelaj et al. | 154 | Squamous cell carcinoma, non-squamous cell carcinoma | Nivolumab or pembrolizumab | 4 | 4.7 months/  2.2 months; N/A | 10.1 months/  2.6 months; N/A |
| [36] | Katayama et al. | 81 | Adenocarcinoma, squamous cell carcinoma, others | Atezolizumab | 5 | 86 days/  42 days;  *p* < 0.001 | Not reached/  98 days;  *p* < 0.001 |
| [37] | Russo et al. | 187 | Squamous cell carcinoma, non-squamous cell carcinoma | Nivolumab | 5 | 7.0 months/  4.0 months; N/A | 15.0 months/  6.0 months;  N/A |
| [38] | Matsubara et al. | 24 | Adenocarcinoma, squamous cell carcinoma, others | Atezolizumab | 5 | N/A | N/A |
| [39] | Takada et al. | 226 | Adenocarcinoma, squamous cell carcinoma, others | Nivolumab or pembrolizumab | 6.05 | N/A | N/A |
| [40] | Ksienski et al. | 220 | Squamous cell carcinoma, non-squamous cell carcinoma | Pembrolizumab | 6.4 | 6.7 months/  2.9 months;  *p* < 0.001 | 18.9 months/  5.4 months;  N/A |
|  | Current study | 236 | Adenocarcinoma, others | Pembrolizumab plus platinum and pemetrexed | 5 | 12.8 months/  5.3 months;  *p* = 0.0002 | 29.4 months/  12.0 months;  *p* < 0.0001 |

All studies included a retrospective design.

NLR, neutrophil-to-lymphocyte ratio; PFS, progression-free survival; OS, overall survival; NSCLC, non-small cell lung cancer; NOS, not specified otherwise; N/A, not available
